# Severe atypical pneumonia in critically ill patients: a retrospective multicenter study

**DOI:** 10.1186/s13613-018-0429-z

**Published:** 2018-08-13

**Authors:** S. Valade, L. Biard, V. Lemiale, L. Argaud, F. Pène, L. Papazian, F. Bruneel, A. Seguin, A. Kouatchet, J. Oziel, S. Rouleau, N. Bele, K. Razazi, O. Lesieur, F. Boissier, B. Megarbane, N. Bigé, N. Brulé, A. S. Moreau, A. Lautrette, O. Peyrony, P. Perez, J. Mayaux, E. Azoulay

**Affiliations:** 1 AP-HP, Medical ICU, Hôpital Saint-Louis, 1 Avenue Claude Vellefaux, 75010 Paris, France; 2 UFR de Médecine, University Paris-7 Paris-Diderot, Paris, France; 3 AP-HP, DBIM, Hôpital Saint-Louis, Paris, France; 4 Hôpital Edouard Herriot, Service de Réanimation Médicale, Hospices Civils de Lyon, Lyon, France; 5 AP-HP, Réanimation médicale, Hôpital Cochin, Paris, France; 6 Réanimation des Détresses Respiratoires et Infections Sévères, Assistance Publique-Hôpitaux de Marseille, Hôpital Nord, Marseille, France; 7 Service de Réanimation, Centre Hospitalier de Versailles, Le Chesnay, France; 8 Department of Medical Intensive Care, CHU de Caen, Caen, France; 9 Service de Réanimation Médicale et Médecine Hyperbare, Hôpital Angers, Angers, France; 10 AP-HP, Medical-Surgical Intensive Care Unit, Avicenne University Hospital, Bobigny, France; 11 Service de Réanimation polyvalente, Angoulême, France; 12 Intensive Care Unit, Draguignan Hospital, Draguignan, France; 13 AP-HP, Groupe Henri Mondor-Albert Chenevier, Service de Réanimation Médicale, Hôpital Henri Mondor, Créteil, France; 14 Service de Réanimation, CH Saint-Louis, La Rochelle, France; 15 AP-HP, Réanimation médicale, Hôpital Européen Georges Pompidou, Paris, France; 16 AP-HP, Department of Medical and Toxicological Critical Care, Lariboisière Hospital, Paris, France; 17 AP-HP, Medical Intensive Care Unit, Hôpital Saint-Antoine, Paris, France; 18 Medical Intensive Care Unit, Centre Hospitalier Universitaire de Nantes, Nantes, France; 19 Centre de réanimation, Hôpital Salengro, CHU-Lille, Lille, France; 20 Service de Réanimation Médicale Polyvalente, CHU Gabriel Montpied, Clermont-Ferrand, France; 21 AP-HP, Service des urgences, Hôpital Saint-Louis, Paris, France; 22 Service de Réanimation médicale, Hôpital Brabois, Nancy, France; 23 AP-HP, Pneumology and Critical Care Medicine Department, Universitary Hospital La Pitié Salpêtrière-Charles Foix, Paris, France

**Keywords:** Pneumonia, Outcome, ICU, *Mycoplasma pneumoniae*, *Chlamydophila pneumoniae*

## Abstract

**Background:**

*Chlamydophila pneumoniae* (CP) and *Mycoplasma pneumoniae* (MP) patients could require intensive care unit (ICU) admission for acute respiratory failure.

**Methods:**

Adults admitted between 2000 and 2015 to 20 French ICUs with proven atypical pneumonia were retrospectively described. Patients with MP were compared to *Streptococcus pneumoniae* (SP) pneumonia patients admitted to ICUs.

**Results:**

A total of 104 patients were included, 71 men and 33 women, with a median age of 56 [44–67] years. MP was the causative agent for 76 (73%) patients and CP for 28 (27%) patients. Co-infection was documented for 18 patients (viruses for 8 [47%] patients). Median number of involved quadrants on chest X-ray was 2 [1–4], with alveolar opacities (*n* = 61, 75%), interstitial opacities (*n* = 32, 40%). Extra-pulmonary manifestations were present in 34 (33%) patients. Mechanical ventilation was required for 75 (72%) patients and vasopressors for 41 (39%) patients. ICU length of stay was 16.5 [9.5–30.5] days, and 11 (11%) patients died in the ICU. Compared with SP patients, MP patients had more extensive interstitial pneumonia, fewer pleural effusion, and a lower mortality rate [6 (8%) vs. 17 (22%), *p* = 0.013]. According MCA analysis, some characteristics at admission could discriminate MP and SP. MP was more often associated with hemolytic anemia, abdominal manifestations, and extensive chest radiograph abnormalities. SP-P was associated with shock, confusion, focal crackles, and focal consolidation.

**Conclusion:**

In this descriptive study of atypical bacterial pneumonia requiring ICU admission, mortality was 11%. The comparison with SP pneumonia identified clinical, laboratory, and radiographic features that may suggest MP or CP pneumonia.

**Electronic supplementary material:**

The online version of this article (10.1186/s13613-018-0429-z) contains supplementary material, which is available to authorized users.

## Background

Severe pneumonia remains the major reasons for admission to the intensive care unit (ICU), mainly related to *Streptococcus pneumoniae* (SP). Atypical pneumonia (AP) related, for instance, to *Chlamydophila pneumoniae* (CP) and *Mycoplasma pneumoniae* (MP) accounts for 1–30% of documented pneumonia in patients admitted to ICU [1–11]. Although AP is rarely severe, some patients with community-acquired AP require ICU admission. Several retrospective studies reported ICU admission for 2–16.3% of patients with AP [1–3, 8, 11–15]. In one study, even 38.8% of patients with AP, older than 65 years, were admitted to ICU [12]. Among ICU-admitted patients with AP, 0.3–11% required mechanical ventilation [4, 5], with acute respiratory distress syndrome (ARDS) for few patients [15, 16]. In previous studies, mortality rates were low, around 3% [5, 13, 14, 17], although a recent retrospective study found 29.4% mortality [12] in a population with high rates of co-infection and cardiac complications.

In previous non-ICU studies, compared to bacterial pneumonia, AP was associated with younger age and fewer comorbidities, a lower risk of severe respiratory failure, and better outcome [4, 6, 13, 14, 18]. For patients admitted to ICU, studies remained rare.

The main objective of the study was to describe AP in patients admitted to ICU. Our secondary objective was to compare the diagnostic strategy and outcomes between *Mycoplasma pneumoniae*-related pneumonia (MP) and *Streptococcus pneumoniae-*related pneumonia (SP) admitted to ICU.

## Methods

### Patients with atypical pneumonia (AP)

This is a retrospective chart review of adults admitted to 20 ICUs in France with a diagnosis of AP over the 16-year period from 2000 to 2015 (Additional file 1: Figure S1). Inclusion criteria were pneumonia defined with sepsis and a new pulmonary infiltrate on the chest radiograph and either a positive specific polymerase chain reaction (PCR) test for MP or CP on respiratory specimens (noninvasive samples or bronchoalveolar lavage) or blood serologic tests suggesting acute MP or CP infection (elevated specific IgM or fourfold increase in IgG level between two time points or elevated anti-MP IgG combined with presence of cold agglutinins) [19].

This study was approved by a local ethic committee (Société de Réanimation de Langue Française, CE SRLF 18-01).

### Data collection

Clinical and laboratory data at ICU admission were collected, as well as organ failure during ICU stay. The SAPS II score [20] was used to assess severity at ICU admission. We also collected extra-pulmonary symptoms; arthritis was defined as new inflammation with one or more joints, myocarditis with cardiac dysfunction and troponin elevation and cutaneous involvement with the onset of skin rash. Bacterial and/or viral co-infections at diagnosis were recorded.

### Patients with *Streptococcus pneumoniae* pneumonia (SP-P)

Patients with MP-AP were compared to a group of consecutive patients with proven SP-P admitted to one of the study ICUs (Saint Louis Hospital, Paris) during the same period. SP-P was diagnosed based on sepsis with a new pulmonary infiltrate and identification of SP in at least one microbiological specimen (blood culture, respiratory specimen, or urinary antigen with no alternative diagnosis).

### Statistical analysis

Categorical variables were described as *n* (%) and quantitative variables as median [25th–75th percentiles]. We first described the features in the patients with AP at ICU admission. Then, we conducted univariate analyses with a nonparametric test to compare the groups with MP-AP and SP-P. Finally, multiple correspondence analysis (MCA) was performed to identify the dimensions associated with the parameters at ICU admission (HIV, symptom duration, shock, confusion, diarrhea, physical chest findings, chest radiograph abnormalities, bilirubin level, and hemolytic anemia) and the causative organism, using the FactoMineR library in the R software platform. MCA is an extension of simple correspondence analysis designed to analyze relations among categorical variables. The aim is to redefine the principal dimensions or axes of the space in a way that captures the highest possible percentage of the inertia (which can be likened to the explained variance).

All tests were two-tailed. *p* values < 0.05 were considered significant. All statistical analyses were carried out using the R 2.13.1 statistical platform (http://www.R-project.org).

## Results

### Clinical findings in the patients with atypical pneumonia (AP)

We included 104 patients, 71 men and 33 women, with a median age of 56 [44–67] years (Additional file 2: Table S2). Acute respiratory failure was the main reason for ICU admission (*n* = 96; 92%); other reasons were cardiovascular failure (*n* = 2), neurological disorders (*n* = 3), and miscellaneous reasons (*n* = 3).

**Fig. 1:**
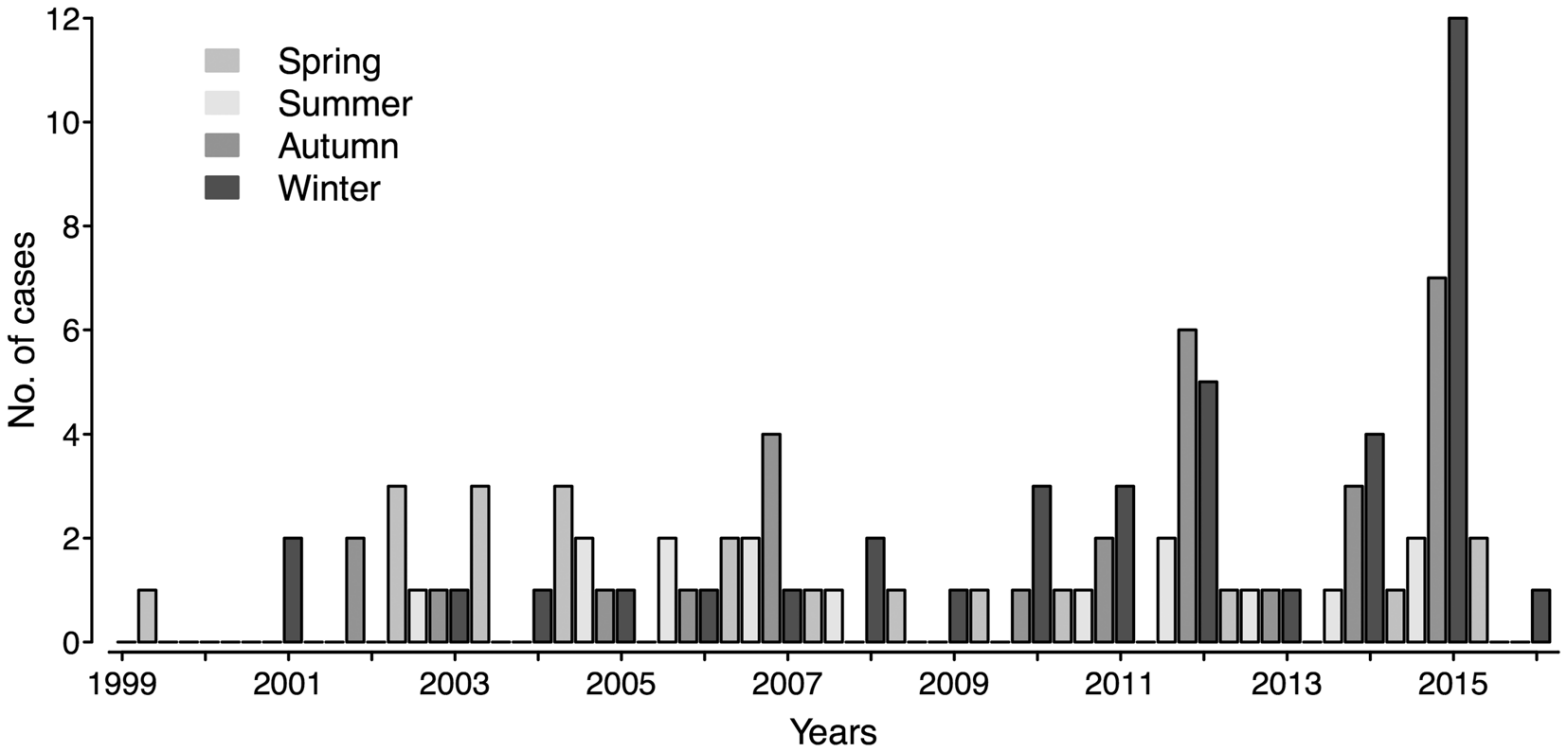
Distribution of atypical pneumonia cases by season and year

AP was more common in the fall and winter (Fig. 1). Furthermore, AP became more common over time, suggesting improved diagnosis after the introduction of PCR testing.

Table 1 and Additional file 3: Table S1 report the main features of the patients with AP. The most common comorbidity was chronic respiratory disease, which was present in 32 (31%) patients including 9 patients with chronic obstructive lung disease, 4 patients with asthma, and 4 patients with interstitial lung disease; of these 32 patients, 7 patients were on long-term oxygen therapy before ICU admission. Immunosuppression was noted in 21 patients including 10 (48%) with hematological malignancies (lymphoma, *n* = 6), 7 with solid cancer, and 2 with HIV infection. Delay from respiratory symptoms onset to ICU admission was 5 [3–8] days. A fever defined with a body temperature above 38.5 °C was present in 77 patients (74%). At ICU admission, all patients were tachypneic (respiratory rate, 32 [26–37]/min) and 48 (46%) had severe respiratory symptoms. Physical chest examination included crackles (*n* = 54; 52%), rhonchi (*n* = 15; 14%), wheezing (*n* = 12; 11%), and signs of consolidation (*n* = 7; 7%). No squeaks were reported. Extra-pulmonary symptoms concerned 34 (33%) patients and included arthritis (*n* = 2), myocarditis (*n* = 4), and skin rash (*n* = 6). Almost one-third of the patients (*n* = 32; 31%) had neurological symptoms at ICU admission, mostly with an altered level of consciousness related to severity of sepsis or to hypoxemia. Confusion was the main symptom for 3 (3%) patients, and meningo-encephalitis was diagnosed in 1 patient. Cold agglutinins assessed in case of hemolytic anemia were positive in 9 (9%) MP patients, cytolysis occurred in 11 (10%) patients, and rhabdomyolysis was present in 3 (3%) patients. At ICU admission, 10 (10%) patients had shock, the SOFA score was 5 [2–7], and the SAPS II was 33 [25–44].

**Table 1:** Clinical characteristics of patients with atypical pneumonia at ICU admission and outcome according to the causative agent

*N* (%) or median [IQR]	Total (*N* = 104)	*Mycoplasma pneumoniae* (*N* = 76)	*Chlamydophila pneumoniae* (*N* = 28)
Demographics			
Age	56 [44–67]	54 [41–69]	64 [52–75]
Female gender	33 (32%)	26 (34%)	7 (25%)
Comorbidities			
Chronic respiratory disease	32 (31%)	22 (29%)	10 (36%)
Current smoker	30 (29%)	20 (38%)	10 (36%)
Immunosuppression	21 (20%)	17 (22%)	4 (14%)
HIV infection	2 (2%)	2 (3%)	0
Hematological malignancy	10 (10%)	9 (12%)	1 (3.5%)
Cancer	7 (7%)	4 (5%)	3 (11%)
Hypertension	32 (31%)	24 (32%)	8 (28%)
Reason for ICU admission			
Acute respiratory distress	96 (92%)	70 (92%)	26 (93%)
Cardiovascular failure	2 (2%)	2 (3%)	0
Neurological disorders	3 3%)	2 (3%)	1 (3.6%)
Other	3 (3%)	2 (3%)	1 (3.6%)
Clinical respiratory findings			
Respiratory rate	32 [26–37]	33 [27–38]	30 [26–33]
Signs of respiratory failure	48 (46%)	33 (49%)	15 (54%)
Rhonchi	15 (14%)	9 (15%)	6 (21%)
Crackles	54 (52%)	36 (47%)	18 (64%)
Signs of consolidation	7 (7%)	5 (9%)	2 (7%)
Decreased vesicular breath sounds	14 (13%)	10 (17%)	4 (14%)
Clinical presentation			
Time since symptom onset (days)	5 [3–8]	6 [4–9]	4 [2–7]
Fever	77 (74%)	58 (83%)	19 (68%)
Shock	10 (10%)	6 (8%)	4 (14%)
Neurological symptoms	32 (31%)	19 (25%)	13 (46%)
Gastrointestinal symptoms	1 (1%)	1 (1%)	0
Extra-pulmonary signs			
≥ 1 extra-pulmonary symptom	34 (33%)	27 (36%)	7 (25%)
Arthritis	2 (2%)	1 (1%)	1 (3.5%)
Myocarditis	4 (4%)	4 (5%)	0
Treatments in the ICU			
Mechanical ventilation	75 (72%)	50 (66%)	25 (89%)
Duration of ventilation	13 [8–19]	12.5 [8–22.5]	13.5 [8.5–19]
Vasopressors	41 (39%)	26 (34%)	15 (54%)
Renal replacement therapy	10 (9.5%)	7 (9%)	3 (11%)
Outcomes			
Death in the ICU	11 (10%)	6 (8%)	5 (18%)
Length of ICU stay (days)			
Discharged alive	15 [8–26]	15 [8–27]	19 [12–24]
ICU death	39 [25–49]	37 [26–47]	39 [25–90]

*HIV* human immunodeficiency virus, *ICU* intensive care unit

### Other findings in patients with atypical pneumonia (AP)

The most common findings by chest radiography were alveolar opacities (*n* = 61, 59%), and interstitial opacities (*n* = 32, 31%) in 2 [1–4] quadrants. Pleural effusion was rare (*n* = 6, 6%).

The causative organism was MP in 76 (73%) patients and CP in 28 (27%) patients and was identified by serological testing (positive IgM or elevated IgG) in 71 patients, positive PCR on respiratory samples in 33 patients (18 on bronchoalveolar lavage, 10 on naso-pharyngeal aspirate, 2 on tracheal aspirate and 4 on nasal swab) and by both diagnostic methods in 5 patients. None of the collected variables differed between patients diagnosed with PCR, serology or both (Additional file 4: Table S3). Co-infection was found in 18 (20%) patients and was related to viruses (*n* = 9; influenza, rhinovirus, respiratory syncytial virus, coronavirus) or bacteria (*n* = 6; *Haemophilus influenzae*, *Proteus mirabilis, Staphylococcus aureus, Serratia marcescens*) or *Pneumocystis jirovecii* (*n* = 3). None of MP patients had co-infection with CP or SP.

### ICU management of atypical pneumonia (AP)

Mechanical ventilation was required for 75 (72%) patients and lasted 13 [8–19] days. Of the 34 (45%) patients meeting criteria for ARDS, 4 required extracorporeal membrane oxygenation. Vasoactive agents were required for 41 (39%) patients, and renal replacement therapy was started for 10 (10%) patients.

The first-line antibiotics were active on MP and CP in 62 (60%) patients. Time from ICU admission to antibiotic initiation was 1 [0–4] day. Combination therapy was used in 61 (59%) patients and consisted to a third-generation cephalosporin (C3G) and a macrolide in 24 (39%) patients, a C3G and a quinolone in 13 (21%) patients, another betalactam and a macrolide in 16 (26%) patients, another betalactam and a quinolone in 6 (9%) patients, or another antibiotic and a macrolide in 2 (3%) patients. Antibiotics was adapted according to microbiology results with a macrolide (*n* = 72), a quinolone (*n* = 24) or a cycline (*n* = 3).

### Outcomes of atypical pneumonia (AP)

Eleven (11%) patients died in the ICU. ICU stay length was 16.5 [9.5–30.5] days. Persistent hypoxemia was present at ICU discharge in 60 (58%) patients. By univariate analysis, factors associated with mortality were age ≥ 65 years (*p *= 0.033), signs of respiratory distress (*p *= 0.017), and interstitial opacities on the chest radiograph (*p *= 0.017). For MP patients, 26 (34%) did not receive adequate antibiotic at ICU admission. Among them 2 patients died.

### Comparison to *Streptococcus pneumoniae* pneumonia (SP-P)

Tables 2 and 3 reports univariate analysis comparing patients with MP-AP and SP-P. Factors significantly associated with SP-P were HIV infection [12 (16%) vs. 2 (3%), *p* = 0.009], neurological symptoms [20 (26%) vs. 1 (1%), *p* < 0.0001], and gastrointestinal symptoms [15 (20%) vs. 1 (1%), *p* = 0.0003]. Factors significantly associated with MP were hemolytic anemia or cold agglutinins [0 (0%) vs. 9 (12%), *p* = 0.003]. Also, 6 patients with SP-P had co-infection (influenza A, *n* = 3; *Haemophilus influenza*e, *n* = 2; *Streptococcus constellatus*, *n* = 1).

**Table 2:** Univariate analysis comparing clinical characteristics and outcomes of patients with *Mycoplasma pneumoniae* versus *Streptococcus pneumoniae* pneumonia

*N* (%) or median (IQR)	Total (*N* = 152)	*Mycoplasma pneumoniae* (*N* = 76)	*Streptococcus pneumoniae* (*N* = 76)	*p* value
Demographics				
Age	55 [43–69]	54 [41–69]	57 [44–73]	0.058
Female gender	51 (34%)	26 (34%)	25 (33%)	1
Comorbidities				
Chronic respiratory disease	36 (24%)	22 (29%)	14 (18%)	0.18
Current smoker	49 (41%)	20 (38%)	29 (43%)	
Immunosuppression	44 (29%)	17 (22%)	27 (36%)	0.11
HIV infection	14 (9%)	2 (3%)	12 (16%)	*0.009*
Hematological malignancy	18 (12%)	9 (12%)	9 (12%)	1
Cancer	12 (8%)	4 (5%)	8 (11%)	0.37
Hypertension	50 (33%)	24 (32%)	26 (34%)	0.86
Reason for ICU admission				
Acute respiratory distress	140 (92%)	70 (92%)	70 (92%)	0.59
Shock	6 (4%)	2 (3%)	4 (5%)	
Neurological symptoms	4 (3%)	2 (3%)	2 (3%)	
Other	2 (1%)	2 (3%)	0	
Clinical respiratory findings				
Respiratory rate	31 [26–38]	33 [27–38]	30 [26–36]	0.43
Signs of respiratory distress	67 (47%)	33 (49%)	34 (45%)	0.74
Rhonchi	21 (16%)	9 (15%)	12 (16%)	1
Crackles	79 (59%)	36 (61%)	44 (59%)	1
Signs of consolidation	27 (21%)	5 (9%)	22 (30%)	*0.008*
Decreased vesicular breath sounds	38 (28%)	10 (17%)	28 (38%)	*0.007*
Clinical presentation				
Time since symptom onset (days)	4 [2–7]	6 [4–9]	3 [2–7]	*0.0008*
Fever	112 (77%)	58 (83%)	54 (71%)	0.12
Shock	30 (20%)	6 (8%)	24 (32%)	*0.0004*
Neurological symptoms	21 (14%)	1 (1%)	20 (26%)	*< 0.0001*
Gastrointestinal symptoms	16 (11%)	1 (1%)	15 (20%)	*0.0003*
Extra-pulmonary signs				
≥ 1 extra-pulmonary sign	66 (43%)	27 (36%)	39 (51%)	0.071
Arthritis	1 (1%)	1 (1%)	0	1
Myocarditis	4 (3%)	4 (5%)	0	0.12
Treatments in the ICU				
Mechanical ventilation	88 (58%)	50 (66%)	38 (50%)	*0.049*
Duration of ventilation (days)				
Discharged alive	11 [7–19]	13 [8–23]	9 [6–16]	
ICU death	11 [3–18]	18 [17–34]	5 [2–15]	
Vasopressors	60 (39%)	26 (34%)	34 (45%)	0.26
Renal replacement therapy	17 (11%)	7 (9%)	10 (13%)	0.49
*SAPS II*	36 [24–47]	32 [22–41]	42 [30–55]	*0.0005*
Outcomes				
ICU stay length (days)				
Discharged alive	9 [5–19]	15 [8–27]	5 [3–10]	
ICU death	13 [4–27]	37 [26–47]	5 [3–14]	
28-day mortality	23 (15%)	6 (8%)	17 (22%)	*0.013*

*HIV* human immunodeficiency virus, *ICU* intensive care unit, *SAPS II* Simplified Acute Physiology Score version II

**Table 3:** Univariate analysis comparing laboratory findings in patients with *Mycoplasma pneumoniae* versus *Streptococcus pneumoniae* pneumonia

*N* (%) or median (IQR)	Total (*N* = 152)	*Mycoplasma pneumoniae* patients (*N* = 76)	*Streptococcus pneumoniae* patients (*N* = 76)	*p* value
Laboratory features				
Lactate (mmol/l)	2.2 [1.6–3.3]	1 [0.7–2.7]	2.3 [1.8–3.4]	0.003
P/F ratio	163 [92–267]	120 [88–236]	178 [114–280]	0.051
Serum sodium (mmol/L)	136 [133–139]	137 [135–140]	136 [132–139]	*0.028*
Creatinine (µmol/L)	87 [65–139.5]	77 [57.5–108]	101 [69.5–168.8]	*0.008*
CPK (IU/l)	122 [40–309]	138 [89–608]	108 [36–202]	0.093
ASAT (IU/l)	38 [23–80]	44 [24–81]	38 [22–77]	0.45
Bilirubin (µmol/l)	12.8 [8–21.7]	8.4 [5.8–13]	15 [9.2–24.5]	*0.0006*
Leukocytes	11,400 [7200–16,300]	11,140 [8100–17,000]	11,200 [5112–16,142]	0.63
Hemoglobin (g/dL)	11.6 [10–12.9]	11.3 [9.6–13.1]	11.6 [10.2–12.8]	0.89
Platelets (Giga/L)	217 [138–287]	262.5 [179.5–311.25]	204 [138–252]	*0.009*
Cytolysis	21 (14%)	8 (11%)	13 (17%)	0.35
Hemolytic anemia/cold agglutinins	9 (6%)	9 (12%)	0	*0.003*
Rhabdomyolysis	5 (3%)	2 (3%)	3 (4%)	1
Radiological features				
Number of quadrants involved				*0.013*
≤ 2	103 (68%)	37 (49%)	66 (87%)	
> 2	25 (16%)	16 (21%)	9 (12%)	
Alveolar opacities	111 (85%)	42 (75%)	19 (68%)	*0.013*
Interstitial opacities	26 (20%)	20 (36%)	12 (43%)	*0.0001*
Pleural effusion	20 (15%)	3 (5%)	3 (11%)	*0.007*

*P/F ratio* ratio of partial pressure of oxygen in arterial blood over fraction of inspired oxygen, *CPK* creatine phosphokinase, *ASAT* aspartate aminotransferase

SP-P was associated with a shorter length of respiratory symptoms before ICU admission (3 days [2–7] vs. 6 days [4–9], *p* = 0.0008). At ICU admission SAPS II score was higher for SP-P (42 [30–55] vs. 32 [22–41], *p* = 0.005), shock was more frequent (32% vs. 8%; *p* = 0.0004), creatinine level was higher (101 [69.5–168.8] μmol/L vs. 77 [57.5–108] μmol/L, *p* = 0.008), and lactate level was high (2.3 [1.8–3.4] mmol/l vs. 1 [0.07–2.7] mmol/l; *p* = 0.003).

Signs of consolidation and decreased breath sounds were more common in SP-P than in MP-AP (30% vs. 9% and 38% vs. 17%, respectively). MP-AP involved 4 quadrants on chest X-ray (26% vs. 8%, *p* = 0.013) but less frequently pleural space (5% vs. 11%, *p* = 0.007). The bilirubin level was higher in the patients with SP-P (15 [9.2–24.5] μmol/L vs. 8.4 [5.8–13] µmol/L, *p* = 0.0006). MP-AP was associated with the use of mechanical ventilation (66% vs. 50%, *p* = 0.049). ICU length of stay (LOS) seemed prolonged in case of MP-AP regardless of the ICU outcome (median LOS 37 vs. 5 days and 15 vs. 5 days, respectively, in patients who died in the ICU and in patients who were discharged alive). However, 28-day mortality was lower in the MP-AP group (5% vs. 20%, *p* = 0.005).

**Fig. 2:**
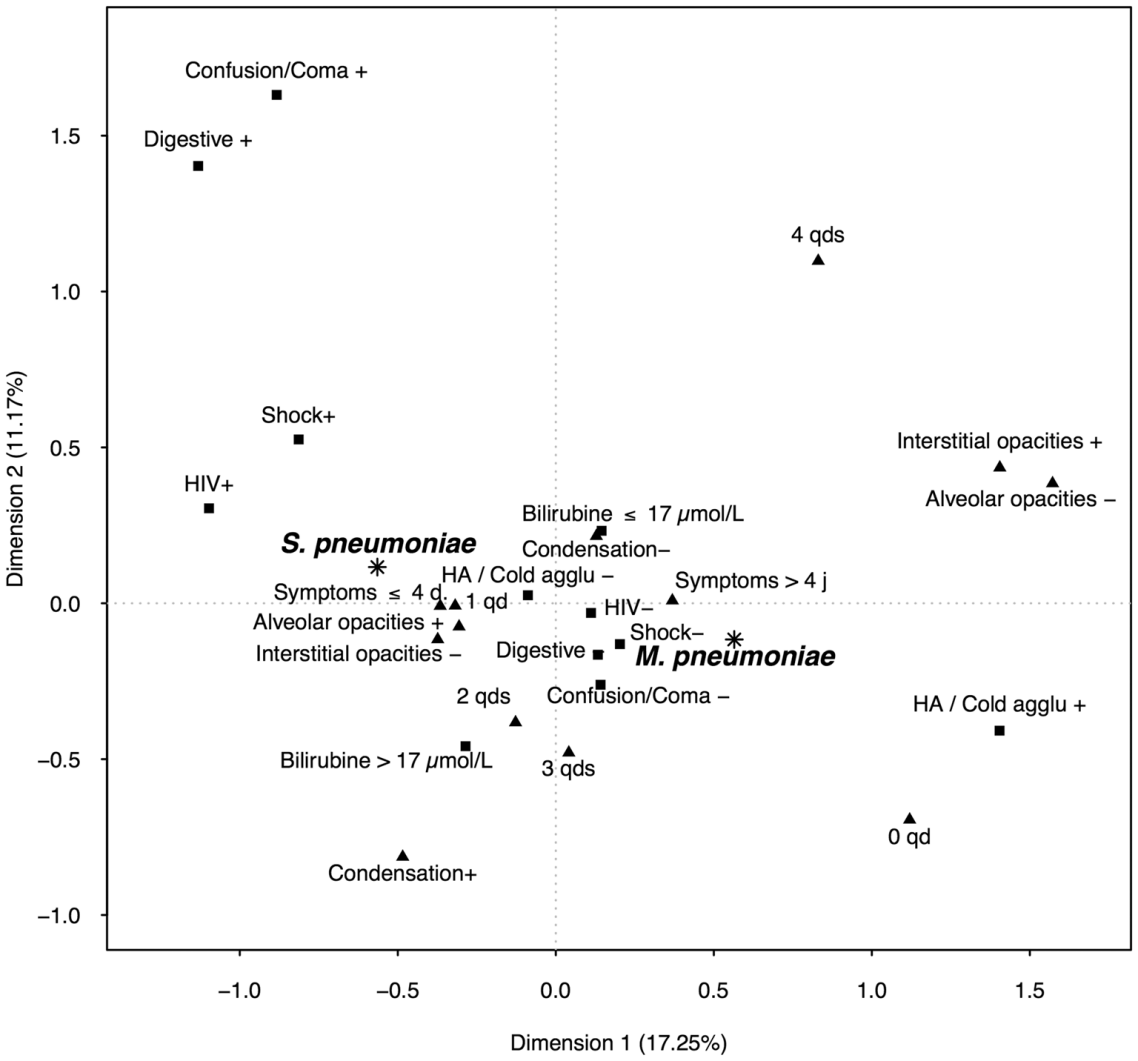
Multiple correspondence analysis: the factors are mapped along two dimensions. Triangles indicate pulmonary signs and squares extra-pulmonary signs. *HA* hemolytic anemia, *Cold agglu+* presence of cold agglutinins, *QD* quadrants

Figure 2 shows the MCA results for the clinical and radiological characteristics at admission. Several characteristics discriminated between MP and CP. MP was more often associated with hemolytic anemia, abdominal manifestations and extensive chest radiograph abnormalities. SP-P was associated with shock, confusion, focal crackles, and focal consolidation.

## Discussion

This multicenter study is the largest one analyzing 104 AP patients admitted to ICU. Extra-pulmonary symptoms were seen for one-third of patients, corresponding to data on previous study for patients not admitted to ICU [21]. However, AP in non-ICU patients was described as mild [6], whereas a substantial proportion of our patients had severe acute pneumonia, with shock at ICU admission for 10% of patients and mechanical ventilation required for 72% of patients including 45% of patients with ARDS.

In previous studies, patients with MP-AP were younger and had fewer comorbidities, lower respiratory disease severity and better outcomes [4, 6, 13, 14, 18]. In our study, with ICU patients, age was similar for patients with MP-AP and SP-P.

Previous studies also compared clinical and radiological features according to the causative organism of pneumonia [8, 15, 18]. In a Japanese cohort, among patients with pneumonia and audible crackles, these were more often heard only in late inspiration in patients with AP and throughout inspiration in patients with other bacteria [22]. In our study, patients with MP-AP had no specific clinical findings, except signs of consolidation which were associated with SP-P. On radiological findings, compared to SP-P, MP-AP was more often responsible for ground-glass opacification, centrilobular nodules, bronchial wall thickening, and diffuse radiological abnormalities [1, 15, 18]. In our study, extensive interstitial pneumonia was more common in MP-AP than in SP-P.

The Japanese Respiratory Society published guidelines for identifying MP-AP [17] and established a scoring system based on six parameters: age < 60 years, minor or no comorbidities, stubborn cough, abnormal chest auscultation, the absence of sputum and of an etiological agent identifiable by rapid diagnostic testing, and peripheral white blood cell count < 10,000/μL. A score ≥ 4 indicates a high probability of MP-AP (sensitivity, 88.7%; and specificity, 77.5%). Another scoring system performed well in separating patients with pneumonia into three groups: pyogenic bacteria; MP, CP, or virus; and unknown agent [23]. Nevertheless, neither scoring system had been assessed in ICU patients. In our study, MCA provided insights into differences between MP-AP and SP-P. Hemolytic anemia, diffuse chest radiograph abnormalities, and interstitial opacities were associated with MP-AP. On the contrary, HIV infection, shock, neurological symptoms, gastrointestinal symptoms, signs of consolidation, shorter symptom duration, higher bilirubin level, and radiological alveolar opacities were strongly linked to SP-P.

Compared to patients with SP-P, those with MP-AP more often required mechanical ventilation and spent more time in the ICU yet had a lower risk of death. This lower mortality may be ascribable to the smaller number of MP-AP patients with extra-pulmonary organ failure (shock, neurological manifestations, acute renal failure) and to the lower SAPS II severity score in the MP-AP group (32 [22–41] vs. 42 [30–55], *p *= 0.005).

Interestingly, intracellular pathogens are underdiagnosed like viruses, but under-covered despite the availability of therapeutic agents. These findings are in line with these from Menendez et al. [24] who reported a lack of antibiotic compliance in patients with CAP. Our descriptive data may be useful to help clinicians to discriminate SP-related pneumonia and MP-related pneumonia, even if a double antibiotherapy active against atypical pathogens is recommended in severe patients.

This study had several limitations. First, the study design was retrospective and patients were included within a 16-year period. ICU management may have changed over this period. ICU admissions criteria could be different according to the center and the year of admission. Atypical pneumonia remains rare, and the main objective of the study was to describe AP in the most severe patients. However, the study assessed mostly the clinical and radiological characteristics at admission which would be unlikely to change between the centers.

Secondarily, SP-P patients were included from only one single center, whereas AP patients were included from several centers. The main objective of the study was to describe patients at ICU admission. Although admission rules would be different between the centers, the clinical presentation would not be affected. Thirdly, only patients with proven AP based on positive microbiologic samples were included. Half of the patients with MP-AP had their diagnosis based on serological testing. More recently only PCR was used to diagnose *Mycoplasma pneumoniae* infection. Positivity of IgM anti-MP is considered as the gold standard, and PCR sensitivity is equal [25]. Although some of the patients had serological tests with fourfold increase in IgG level between two time points, we believe that we included only proven MP-AP patients. Although different diagnostic tests were used within the study period and among the centers, those tests were enough sensitive and specific to include real MP-AP pneumonia.

Fourth, we did not include patients with *Legionella pneumophila* pneumonia, a more frequent atypical pneumonia. Although *Legionella pneumophila* pneumonia was associated with higher risk of ICU admission comparing to MP-AP and CP-AP, our goal was to focus on AP that is usually non-severe and only occasionally leads to ICU admission. Moreover, several studies analyzed *Legionella pneumophila* pneumonia. Similarly, we did not include more rare etiology of pneumonia as Q fever.

## Conclusion

Although considered as less severe pneumonia, atypical pneumonia requiring ICU admission remained associated with 11% mortality. At ICU admission, several clinical and radiological features could differ between MP-AP and SP-P, which may help physicians. Prospective studies are needed to validate clinical model to AP in ICU patients.

## Additional files

**Additional file 1: Figure S1.** Flow chart: ICU admission and diagnostic strategy.

**Additional file 2: Table S2.** Cases distribution (MP and CP-AP patients) by center.

**Additional file 3: Table S1.** Laboratory findings in patients with atypical pneumonia according to the causative agent.

**Additional file 4: Table S3.** Clinical characteristics of patients with atypical pneumonia at ICU admission and outcome according to the diagnostic methods.

## Abbreviations

AP: atypical pneumonia
ARDS: adult respiratory distress syndrome
CP: *Chlamydophila pneumoniae*
ICU: intensive care unit
MCA: multiple correspondence analysis
MP: *Mycoplasma pneumoniae*
MP-AP: atypical pneumonia due to *Mycoplasma pneumoniae*
PCR: polymerase chain reaction
SAPS II: Simplified Acute Physiology Score version II
SP: *Streptococcus pneumoniae*
SP-P: pneumonia due to *Streptococcus pneumoniae*
