# Supplementary material for: Severe atypical pneumonia in critically ill patients: a retrospective multicenter study
Source: Ann Intensive Care. 2018 Aug 13;8:81. doi: 10.1186/s13613-018-0429-z (PMC6089852; doi:10.1186/s13613-018-0429-z)
Supplement: Supplementary file 1 — Additional file 1: Figure S1. Flow chart: ICU admission and diagnostic strategy. [file 13613_2018_429_MOESM1_ESM.docx]

**Figure S1. Flow chart: ICU admission and diagnostic strategy**

**Patients with acute community-acquired pneumonia needing ICU admission** (between 2000 and 2015, 20 ICU centers)

**N = 33765**

**Atypical pneumonia (*Mycoplasma pneumoniae* or *Chlamydophila pneumoniae*)**

**N = 104**

***Mycoplasma pneumoniae* patients**

**N = 76**

***Chlamydophila pneumoniae* patients**

**N = 34**

**Positive serology**

**N = 43/45**

**Positive PCR**

**N = 35/35**

**Positive PCR**

**N = 3/3**

**Positive serology**

**N = 28/28**
