# Supplementary material for: Severe atypical pneumonia in critically ill patients: a retrospective multicenter study
Source: Ann Intensive Care. 2018 Aug 13;8:81. doi: 10.1186/s13613-018-0429-z (PMC6089852; doi:10.1186/s13613-018-0429-z)
Supplement: Supplementary file 2 — Additional file 2: Table S2. Cases distribution (MP and CP-AP patients) by center. [file 13613_2018_429_MOESM2_ESM.docx]

| **Center** | **Total**  **(N = 104)** | | ***Mycoplasma pneumoniae***  **(n = 76)** | ***Chlamydophila pneumoniae***  **(n = 28)** |
| --- | --- | --- | --- | --- |
| Cochin, Paris  Marseille  Lyon  Versailles  Saint Louis, Paris  Angers  Caen  Avicenne, Bobigny  Draguignan  Henri Mondor, Créteil  Nantes  HEGP, Paris  Pitié Salpétrière, Paris  Lariboisière, Paris  Saint Antoine, Paris  La Rochelle  Angoulême  Nancy  Lille  Clermont Ferrand | 13  9  14  9  6  6  6  5  5  4  3  3  1  3  3  4  5  1  2  2 | | 10  9  9  7  6  6  6  4  4  4  3  2  1  1  1  1  1  1  0  0 | 3  0  5  2  0  0  0  1  1  0  0  1  0  2  2  3  4  0  2  2 |
|  | |  |  |  |

**Supplemental Table S2.** Cases distribution (MP and CP-AP patients) by center
