# Supplementary material for: Severe atypical pneumonia in critically ill patients: a retrospective multicenter study
Source: Ann Intensive Care. 2018 Aug 13;8:81. doi: 10.1186/s13613-018-0429-z (PMC6089852; doi:10.1186/s13613-018-0429-z)
Supplement: Supplementary file 3 — Additional file 3: Table S1. Laboratory findings in patients with atypical pneumonia according to the causative agent. [file 13613_2018_429_MOESM3_ESM.docx]

**Supplemental Table S1.** Laboratory findings in patients with atypical pneumonia according to the causative agent

| **N (%) or Median (IQR)** | **Total**  **(N = 104)** | | | ***Mycoplasma pneumoniae***  **(N=76)** | | ***Chlamydophila***  ***pneumoniae***  **(N=28)** |
| --- | --- | --- | --- | --- | --- | --- |
| **Laboratory features**  P/F ratio  Serum sodium (mmoL/L)  Creatinine (μmoL/L)  CPK (UI/l)  ASAT (UI/l)  Bilirubin (µmol/l)  Leukocytes  Hemoglobin (g/dL)  Platelets (Giga/L)  Cytolysis  Rhabdomyolysis  **Radiological features**  Number of quadrants involved  0  1  2  3  4  Alveolar opacities  Interstitial opacities  Pleural effusion | 145 [89-242]  137 [134-139]  85 [63-122]  183 [77-573]  53 [32-98]  9.75 [6-14.5]  11800 [8200-16870]  11.8 [9.9-13.5]  242 [141-303]  11 (10.5%)  3 (3%)  3 (3%)  22 (21%)  31 (30%)  3 (3%)  28 (27%)  61 (59%)  32 (31%)  6 (6%) | | | 120 [88-236]  137 [135-140]  77 [57.5-108]  138 [89-608]  44 [24-81]  8.4 [5.8-13]  11140 [8100-17000]  11.3 [9.6-13.1]  262.5 [179.5-311.25]  8 (11%)  2 (3%)  2 (4%)  17 (32%)  18 (34%)  2 (4%)  14 (26%)  42 (75%)  20 (36%)  3 (5%) | | 176 [75-240]  134 [130-137]  113 [83-146]  301 [65-408]  73 [37-114]  14 [8.75-17.5  12895 [10200-15120]  13 [10.7-13.9]  209 [141-259]  3 (11%)  1 (3.5%)  1 (3.5%)  5 (18%)  3 (11%)  1 (3.5%)  14 (50%)  19 (68%)  12 (43%)  3 (11%) |
|  | |  |  | |  | |

P/F ratio, ratio of partial pressure of oxygen in arterial blood over fraction of inspired oxygen; CPK, creatine phosphokinase; ASAT, aspartate aminotransferase
