# Supplementary material for: Severe atypical pneumonia in critically ill patients: a retrospective multicenter study
Source: Ann Intensive Care. 2018 Aug 13;8:81. doi: 10.1186/s13613-018-0429-z (PMC6089852; doi:10.1186/s13613-018-0429-z)
Supplement: Supplementary file 4 — Additional file 4: Table S3. Clinical characteristics of patients with atypical pneumonia at ICU admission and outcome according to the diagnostic methods. [file 13613_2018_429_MOESM4_ESM.docx]

**Table S3:** Clinical characteristics of patients with atypical pneumonia at ICU admission and outcome according to the diagnostic methods

| **N (%) or Median [IQR]** | **Positive serology only**  **N = 66/71** | **Positive PCR only**  **N =33/38** | **Positive serology and PCR**  **N = 5/7** | **p value** |
| --- | --- | --- | --- | --- |
| **Demographics**  Age  **Comorbidities**  Current smoker  Immunosuppression (not AIDS)  HIV infection  Hypertension  Diabetes  **Clinical respiratory findings**  Respiratory rate  Signs of respiratory distress  Rhonchi  Crackles  Signs of consolidation  **Clinical presentation**  Time since symptom onset (days)  Fever  Shock  Neurological symptoms  **SAPS II**  **Extrapulmonary signs**  ≥1 extrapulmonary symptom  Myocarditis  **Treatments in the ICU**  Mechanical ventilation  Vasopressors  Renal replacement therapy  **Outcomes**  Death in the ICU  Length of ICU stay (days) | 56 [44-58]  19 (40%)  9 (13%)  1 (1.5%)  19 (28%)  9 (13%)  31 [27-27]  30 (50%)  11 (19%)  34 (60%)  3 (6%)  6 [4-9]  50 (80%)  4 (6%)  22 (33%)  32 [23-44]  24 (35%)  1 (1.5%)  50 (73%)  26 (39%)  6 (9%)  9 (13%)  17.5 [10-25.5] | 59 [42-67]  9 (40%)  10 (33%)  1 (3.3%)  11 (37%)  6 (20%)  33 [25-35]  15 (53%)  3 (12%)  18 (75%)  3 (12%)  4.5 [2-7]  22 (78%)  5 (6%)  9 (31%)  35 [27-42]  9 (30%)  2 (6.6%)  21 (70%)  13 (43%)  3 (10%)  1 (3.3%)  15 [7-33] | 56 [33-63]  2 (66%)  0  0  2 (33%)  1 (16%)  32 [24-34]  3 (50%)  1 (25%)  2 (40%)  1 (20%)  7 [4.5-7.5]  5 (100%)  1 (20%)  1 (20%)  33 [29-41]  1 (16%)  1 (16%)  4 (66%)  2 (40%)  1 (20%)  1 (16%)  21 [8-90] | 0.74  0.81  0.03  0.73  0.71  0.69  0.95  0.95  0.73  0.25  0.33  0.21  0.52  0.19  0.82  0.86  0.60  0.11  0.89  0.12  0.93  0.30  0.85 |

HIV, human immunodeficiency virus; ICU, intensive care unit
